# Prevalence of enteric parasites in patients with gastrointestinal symptoms attending a tertiary care center in Central Kerala

**DOI:** 10.3205/dgkh000640

**Published:** 2026-03-10

**Authors:** Keerthy Bose, Joana Magdaline

**Affiliations:** 1 Department of Microbiology, Government Medical College, Kollam, India; 2 Department of Microbiology, Government Medical College, Ernakulam, India

**Keywords:** enteric parasites, gastrointestinal symptoms, coccidian enteric parasites, prevalence, Central Kerala, concentration technique

## Abstract

**Background:**

Intestinal parasitic infections are distributed throughout the world, but with greater prevalence in low socio-economic communities in the tropics and subtropics. Parasitic infections can manifest as an asymptomatic carrier state, gastrointestinal symptoms, or surgical problems. Few studies have been conducted on the prevalence of these infections in Kerala. Thus, this study aimed to estimate the proportion of enteric parasites in stool samples of patients with gastrointestinal symptoms attending a tertiary care center in Central Kerala.

**Methods:**

A total of 280 stool samples from patients at the Government Medical College center were included in study. In the Department of Microbiology, the prevalence of intestinal parasitic infections was detected by microscopic examination of stool specimens. Stool samples were subjected to the formol-ether concentration technique (FECT), followed by modified hot Ziehl-Neelsen staining and fluorescent staining. The results were recorded, and data were presented as frequency and distribution.

**Results:**

Out of 280 patients, parasitic infection was present in 2.86% (n=8). Overall, helminthic infections (66.67%) were more common than protozoal infections (33.33%). The most prevalent parasites were *Ascaris* *lumbricoides* (0.71%) and *Strongyloides stercoralis* (0.71%). The other parasites present were *Entamoeba histolytica, Giardia lamblia, Cryptosporidium parvum, Enterobius vermicularis* and *Trichuris trichiura*. The association between presence of immunosuppressive conditions and presence of parasites in stool was found to be statistically significant.

**Conclusion:**

This study highlights the importance of including stool concentration methods as part of routine diagnostic tests for parasites. The significant association between immunosuppresion and the presence of intestinal parasites should be borne in mind.. Awareness about the importance of personal hygiene, safe water supply, and health education should be given to target populations.

## Zusammenfassung

**Hintergrund:**

Intestinale Parasitosen sind weltweit verbreitet, wobei sie in tropischen und subtropischen Regionen mit niedrigem sozioökonomischem Status häufiger auftreten. Parasitosen äußern sich in Form von asymptomatischer Trägerschaft, gastrointestinalen Symptomen oder chirurgischen Problemen. Bislang gibt es nur wenige Studien zur Prävalenz dieser Erkrankungen in Kerala. Ziel dieser Studie war es, den Anteil von Darmparasiten in Stuhlproben von Patienten mit gastrointestinalen Symptomen zu ermitteln, die das tertiäre Versorgungszentrum in Zentral-Kerala aufsuchten.

**Methoden:**

Insgesamt wurden 280 Stuhlproben von Patienten, die in der Abteilung für Mikrobiologie des Government Medical College in Ernakulam aufgenommen wurden, in die Studie einbezogen. Die Prävalenz von Darmparasitosen wurde durch mikroskopische Untersuchung der Stuhlproben ermittelt. Die Stuhlproben wurden einer Formol-Ether-Konzentrationstechnik (FECT) unterzogen, gefolgt von einer modifizierten Ziehl-Neelsen-Heißfärbemethode und einer Fluoreszenzfärbung. Die Ergebnisse wurden aufgezeichnet und die Daten in Form von Häufigkeit und Verteilung dargestellt.

**Ergebnisse:**

Von 280 Patienten wiesen 2,9% (n=8) eine parasitäre Infestation auf. Insgesamt waren Helmintheninfektionen (66, 7%) häufiger als Protozoeninfektionen (33,3%). Die am häufigsten vorkommenden Parasiten waren *Ascaris lumbricoides* (0,7%) und *Strongyloides stercoralis* (0,7%). Die anderen vorhandenen Parasiten waren *Entamoeba histolytica, Giardia lamblia, Cryptosporidium parvum, Enterobius vermicularis* und *Trichuris trichiura*.

**Schlussfolgerung:**

Die Studie unterstreicht die Bedeutung der Einbeziehung von Methoden zur Stuhlkonzentration als Teil der routinemäßigen Diagnosetests auf Parasiten. Auch bei immunsupprimierten Patienten ist die Untersuchung auf Darmparasiten von Bedeutung. Die Zielgruppe sollte für die Bedeutung der persönlichen Hygiene, einer sicheren Wasserversorgung und Gesundheitsaufklärung sensibilisiert werden.

## Introduction

Intestinal parasitoses are one of the major public health problems in several developing countries, including India. It is estimated that 60% of the world population is infested with enteric parasites [1], with greater prevalence in low socio-economic communities in the tropics [2]. 

Amoebiasis, giardiasis, ascariasis, hookworm infection and trichuriasis are the most common intestinal parasitic infection worldwide [3]. In India, the prevalence of intestinal parasitic infestation ranges from 12.5% to 66%, with varying rates for individual parasites [4], [5]. The risk factors for the greater prevalence of such infections in India include a humid climate, malnutrition, insanitary environments, improper and unsafe sewage and human waste disposal, and low standards of personal hygiene [6]. 

In Kerala, the prevalence of parasitic infections is low because of the implementation of good healthcare services, school health programs and community interventions directed at vulnerable groups [7]. Highly-effective, safe single-dose drugs, such as albendazole, are available for management of parasitic infections are dispensed through the above programs and services.

Parasitic infections can manifest as an asymptomatic carrier state, gastrointestinal symptoms, manifest as surgical emergencies or require surgical intervention due to the mechanical, obstructive, or inflammatory damage they cause to host tissues [6]. Gastrointestinal parasitic infestations that cause self-limited diarrhoea in immunocompetent patients may cause profuse diarrhoea in immunocompromised patients [8]. Many of the opportunistic parasites, e.g., cryptosporidium, cyclospora and cystoisospora, exert their most devastating effects in patients with acquired or congenital immunodeficiencies [8], [9]. 

Proper laboratory investigations into the parasitic etiology of diarrhea lead to prompt and effective management of patients, thereby reducing the morbidity and mortality. Such investigations also help to prevent emergence of drug resistance by irrational use of antibacterial agents to manage diarrhea.

There is a paucity of data from Kerala regarding the epidemiological pattern of enteric parasites. Moreover, the epidemiological pattern of these parasites varies in different geographical regions. This prompted us to conduct a study on detecting the prevalence of enteric parasites among patients in this geographic area with special emphasis on opportunistic enteric parasites.

## Materials and methods

The study was conducted from January 2018 to December 2018 at the Department of Microbiology, Government Medical College, Ernakulam, Kerala. A total of 280 samples collected from patients with symptoms suggestive of parasitic infections attending our hospital were included in the study. Samples were collected in wide-mouthed clean, dry, screw capped, labelled plastic containers and examined within 1–2 hours of collection. Macroscopic examination was done for color, consistency, presence of adult worms or helminthic body segments, as well as the presence of blood or mucus. All stool samples underwent direct microscopic examination (saline wet mount and iodine wet mount) and formalin-ether concentration for protozoan cysts, oocysts, helminthic eggs. The sediment obtained after concentration was again subjected to saline and iodine wet-mount examination to screen for cysts of protozoa as well as larvae and eggs of helminths that might have been missed in direct wet-mount examination. From the sediment obtained, two fecal smears were prepared from each sample, air-dried and fixed in methanol. One smear was stained by modified Ziehl-Neelsen staining and other stained by auramine-phenol fluorescent staining. Although cold and hot methods for modified Ziehl-Neelsen staining were found to be equally effective, the hot method was performed as it is less expensive than the cold method. Fecal smears were stained with strong carbol fuchsin, heated, and allowed to act for 5 minutes. They were decolorized in 1% sulphuric acid for 30 seconds. Then, they were counterstained with methylene blue for 1 minute. These special staining techniques were performed for the detection and identification of coccidian enteric parasites such as cryptosporidium, cyclospora and cystoisospora, which are difficult to identify in routine wet-mount preparation. 

The centrifuged deposit of formol-ether sedimentation was also used to perform auramine-phenol fluorescent staining. Methanol-fixed smears were stained with auramine-phenol solution for 15 minutes. They were then decolorized with 0.5% acid-alcohol for 2 minutes. Smears were counterstained with 0.5% KMnO_4_ solution for 2 minutes.

The data entered in the MS-Excel spreadsheet were analyzed using Statistical Package for Social Sciences (SPSS) software 16.0. Qualitative variables were summarized using frequency or percentage. The chi-squared test was used to analyze study variables. The level of statistical significance was set at p<0.05.

## Results

A total of 280 stool samples were screened for parasites. Out of all the samples examined, 8 (2.8%) were found to be positive for at least one parasite. One patient had dual parasitic infestation. In general, helminthic infections (66.7%) were more prevalent than protozoa (33.33%). 

Males showed a higher prevalence rate of 75% for intestinal parasites than did females (25%) (Table 1 (Tab. 1)). A greater number of samples were received from in-patients (166), of whom 7 tested positive (4.2%). Natives constituted the main study population (91.4%); 24 migrants (8.6%) were also included in the study. The proportion of parasitic ova, larvae, cysts and worms were found to be higher in the migrant population (8.3%).

The parasites detected includes *Entamoeba histolytica, Giardia lamblia, Cryptosporidium parvum, Ascaris lumbricoides, Trichuris trichiura, Enterobius vermicularis* and *Strongyloides stercoralis*. Of these, the most common type were *Strongyloides stercoralis* and *Ascaris lumbricoides *(0.7% each) (Table 2 (Tab. 2)).

Trophozoites and cysts of *Giardia lamblia* were observed in the stool sample of the same patient. One coccidian parasite (*Cryptosporidium parvum*) was obtained and detected using modified Ziehl-Neelsen staining and confirmed by auramine-phenol fluorescent microscopy. One patient presented with dual infection; fertilized and unfertilized eggs of *Ascaris lumbricoides* and eggs of *Trichuris trichiura* were detected in the same stool sample.

Out of the 178 rural patients screened in our study, 7 tested positive (3.9%), while among 102 urban patients tested, only one case was found to be positive (1%) for enteric parasites. 40 patients had conditions associated with immunosuppression (14.3%). Diabetes mellitus was the major factor among the 40 patients (60%). Among the 8 positive cases, 50% had conditions associated with immunosuppression (Table 3 (Tab. 3)). The association between presence of immunosuppressive conditions and presence of parasites in stool was found to be statistically significant. 

Parasites detected in immunocompromised cases were* Strongyloides stercoralis, Entamoeba histolytica* and *Cryptosporidium parvum* (Table 4 (Tab. 4)). Larvae of *Strongyloides stercoralis* were detected from 2 patients with immunosuppresssion, of whom one patient was on long-term steroid therapy for vasculitis management. Both had had diabetes mellitus for the past 10 years. Oocysts of *Cryptosporidium parvum* were detected from the stool sample of one patient who had a history of diabetes mellitus and steroid intake. Oocysts were identified by modified Ziehl-Neelsen staining and fluorescent staining of concentrated stool samples. There was a statistically significant association between presence of diabetes mellitus, immunosuppressive conditions, and presence of parasites in stool.

## Discussion

Among the total samples tested, 8 (2.9%) were positive for parasitic ova/cysts/trophozoites/worms/larvae. Studies from different parts of the world showed different prevalence rates for enteric parasitic infection. The prevalence was found to be 3.9% in a study conducted in Nigeria in 2010 [10], 6.6% in a study conducted in Central India in 2012 [11], and 5.6% in a similar study done in Western India, which were all comparable to that obtained in our study [3]. Another study done in Kenya in 2015 and in rural Bihar in 2015 reported enteric parasitic prevalence as high as 46.5% and 40.3%, respectively [12], [4].

The prevalence of enteric parasitic infection may vary globally depending upon the epidemiological and geographical factors, including time and place of study [13]. The low prevalence obtained in our study may be due to decreased risk factors associated with enteric parasitic infections, like that explained in a comparable study conducted by Davane et al. in Maharashtra [14]. The risk factors listed in the study include unsafe drinking water supply, open air defecation practices, no hand washing after defecation, no wearing of footwear. The government also plays an important role by implementing programs such as Swachh Bharat Mission, launched on October 2, 2014, as a nation-wide campaign [15]. The low prevalence may also be because of initial treatment of patients at primary and secondary healthcare centers before being referred to our tertiary hospital.

The most common class of parasites detected in our study were helminths (2.1%), with the least common being protozoa (1.07%). This finding is similar to that of Rehana et al. [5], also conducted in a tertiary care hospital, Western U.P. in 2016, with the objective of studying intestinal parasites. Helminthic infection was detected in 20.7% cases, while protozoal infection was found in 15.4% cases [5]. In contrast, astudy by Manochitra et al. [1] in Puducherry in 2011 found that protozoan infections (16.3%) were higher than helminthic infections (6%) [1]. This may be due to differences in distribution of parasites depending on epidemiological factors.

*Ascaris lumbricoides* and *Strongyloides stercoralis* were the most common parasites isolated (0.71%) in our study. Considering similar studies done in Nigeria by Olusegun et al. [13] and in Maharashtra by Davane et al. [14], Ascaris lumbricoides infestation was the most common overall. However, different studies conducted globally as well as nationally show different patterns of parasitic prevalence, in which many studies reported the most common parasites to be *Entamoeba histolytica, Giardia lamblia*, and *Ascaris lumbricoides* [4], [16]. In addition, studies that compare the case detection rates in one area at different times, sampling a similar group of patients, indicate that the etiology of infection may change over time [17].

This study found a statistically significant association between presence of immunosuppressive conditions and presence of parasites in stool. 14.3% (n=40) of the study population had history of conditions associated with immunosuppression, e.g., diabetes mellitus, cancer, HIV, history of immunosuppressant consumption for underlying medical illnesses, among which diabetes mellitus constituted the major risk factor (60%). Out of the 40 immunosuppressed patients tested, 10% (n=4) were positive for parasitic infestation. All of them had diabetes mellitus. There was also a statistically significant association between the presence of diabetes mellitus, immunosuppressive conditions, and parasites in stool. This may be due to the disturbance in cellular and humoral immune mechanisms that aids in the elimination of pathogens. Bora et al. [8] made a comparable observation in their study on intestinal parasites among immunosuppressed patients in Meghalaya; there, enteric parasitic infections were present in 53%. The most common parasites seen in their study were *Entamoeba histolytica* and *Ascaris lumbricoides*, as opposed to the present study, in which *Strongyloides stercoralis* was the major parasite detected among immunocompromised patients.

In our study, we examined wet-mount preparations after formol-ether concentration for detection of parasites. We found that the formol-ether sedimentation method is more sensitive than direct stool wet-mount examination. The concentration method could detect 3.2% of parasites, whereas direct examination could detect only 2.1% of parasites. This agrees with different studies conducted globally and nationally [8], [18]. A study conducted in Nigeria [19] comparing the two techniques had congruent results. The formol-ether concentration technique detected 65.3% positive samples, whereas direct examination only 34.7%. The formol-ether concentration technique should be adopted for routine fecal examination, since it detects a higher percentage of infection missed by direct examination, which is the usual method of parasitic examination employed by medical diagnostic laboratories and hospitals in many developing countries.

Special techniques were also applied in this study to detect coccidian enteric parasites that may be overlooked in routine microscopic examination. Fluorescent microscopic examination using auramine phenol and the modified Ziehl-Neelsen technique was used. This was able to detect a case of cryptosporidiosis in our study. A different study was done to compare the sensitivity and specificity of these two methods in the detection of coccidian enteric parasites; results of that study were also slightly different with regard to these parameters [19]. Since the sensitivity and specificity of the two staining methods were comparable, the choice of method must be based on other criteria, such as cost, time, and ease [19]. Most of the studies use only one of the two methods for parasite detection [9], [20], [21]. Since we performed both tests in this study, the results are expected to be more accurate. 

## Conclusions

This study was conducted to determine the prevalence of parasites in patients with gastrointestinal symptoms at the Government Medical College hospital in Ernakulam. Among the total samples tested, 8 (2.9%) were positive for parasites. The occurrence of intestinal parasitic infections was low, and intestinal helminths were more common than other parasites. The study demonstrated a statistically significant association between immunosuppressive conditions and the presence of enteric parasites. The formol-ether sedimentation method was found to be more sensitive than direct wet-mount microscopic examination in the detection of enteric parasites. Understanding the burden of enteric parasites emphasizes their public health importance and helps to implement control measures. Early diagnosis and treatment, health education, safe water supply and sanitation services play a pivotal role in controlling these infections in developing and under-developed nations.

## Notes

### Competing interests

The authors declare that they have no competing interests.

### Author’s ORCID

Bose K: https://orcid.org/0009-0008-0116-9007

### Ethical approval 

The study was approved by the ethics committee of Government Medical College, Ernakulam (Registration number Kerala State Medical Council of authors – Dr. Keerthy Bose – 48980, Dr. Joana Mary Magdaline – 16820).

### Funding

None. 

## Figures and Tables

**Table 1:**
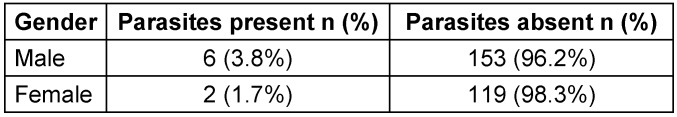
Distribution of parasites among males and females

**Table 2:**
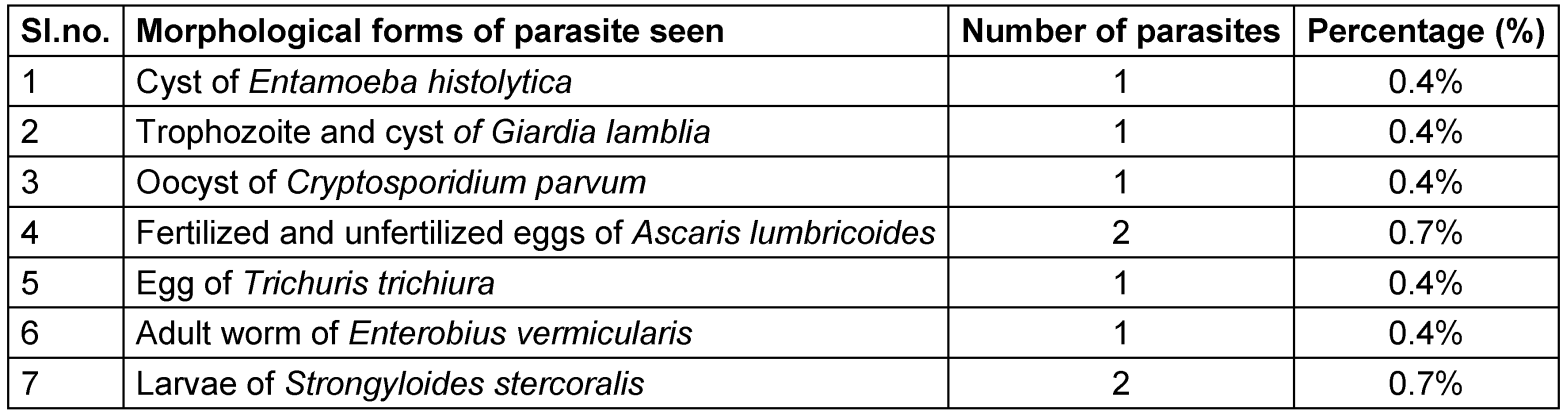
Distribution of different morphological forms of parasites isolated

**Table 3:**
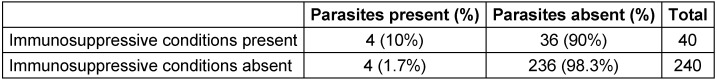
Proportion of parasitic infection in immunocompromised patients

**Table 4:**
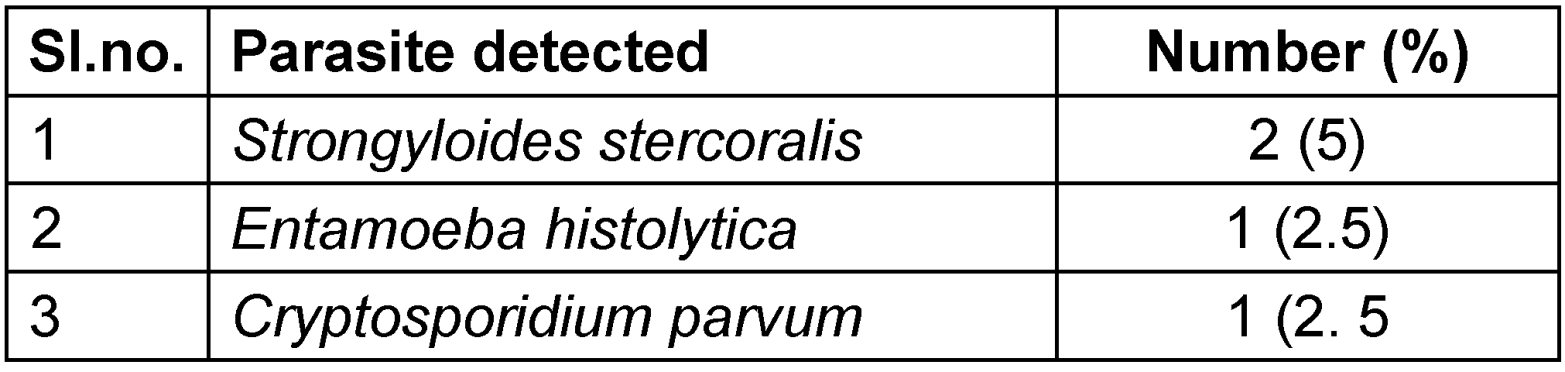
Parasites detected among enteric-parasite-positive patients with immunosuppression
